# What is neuromorphic about neuromorphic computing?

**DOI:** 10.3389/fnins.2026.1980751

**Published:** 2026-10-14

**Authors:** Shantanu Chakrabartty, Chetan S. Thakur, Andreas G. Andreou

**Affiliations:** 1 Department of Electrical and Systems Engineering, Washington University in St. Louis, Saint Louis, MO, United States; 2 Department of Electronic Systems Engineering, Indian Institute of Sciences, Bengaluru, India; 3 Department of Electrical Engineering, The Johns Hopkins University, Baltimore, MD, United States

**Keywords:** artificial intelligence, computing, energy-efficiency, neuromorphic engineering, thermodynamics

By any bibliometric measure, neuromorphic computing is booming. Publications carrying the label have grown steadily for over a decade with proliferation of dedicated journals, workshops, and funding programs. A recent community roadmap has declared the field ready for deployment “at scale” (Kudithipudi et al., 2025). The hardware has kept pace with the literature. Examples include IBM's TrueNorth integrated a million digital spiking neurons on a single die (Merolla et al., 2014); Intel's Loihi processors lie at the core of Hala Point, a system exceeding a billion neurons (Davies et al., 2018); SpiNNaker and BrainScaleS have matured into second-generation, wafer-scale and supercomputer-scale platforms (Furber et al., 2014; Pehle et al., 2022); and memristive crossbar arrays promise in-memory analog computation (Chua, 1971; Strukov et al., 2008). Startups, national initiatives, and semiconductor roadmaps now routinely list “neuromorphic” among the post-Moore alternatives.

Yet beneath this boom lies an awkward foundational question: what, exactly, is neuromorphic? Classical computing rests on a crisp abstraction, namely, Boolean logic, the Turing machine, the von Neumann architecture. This abstraction is entirely indifferent to whether it is realized in relays, vacuum tubes, or emerging fabrication technologies. Quantum computing likewise has a precise essence: state vectors in Hilbert space, unitary evolution, entanglement, and measurement, a definition sharp enough to support rigorous claims of quantum advantage (Arute et al., 2019). Neuromorphic computing has no comparable statement of principle. “Brain-inspired” feels like a family resemblance, not a definition. The field itself has begun to sense this and a wave of recent editorials and perspective articles now asks what the essence of neuromorphic or *neuromorphicism*. Most pointed is Indiveri's editorial “Neuromorphic is dead. Long live neuromorphic”, which argues that the label has drifted far from its founding meaning (Indiveri, 2025).

History clarifies what the early pioneers of the field intended. Carver Mead coined “neuromorphic” electronic systems (Mead, 1990), in the late 1980s to describe circuits that exploit the physics of transistors operated in the subthreshold regime, where channel current depends exponentially on gate voltage in close analogy to ionic conductances in neuronal membranes. His 1990 manifesto argued that biology computes with 10,000-fold to billion-fold less energy than digital logic precisely because it uses device physics rather than fighting it (Mead, 1990). John Hopfield showed that computation could be a collective, emergent property of a dynamical system relaxing in an energy landscape (Hopfield, 1982), Sejnowski and Hinton introduced statistical mechanics into neural computation (Ackley et al., 1985), contributions that were in retrospect worthy of a Nobel Prize in Physics (Royal Swedish Academy of Sciences, 2024). Richard Feynman, who taught the famous course on the physics of computation alongside Mead and Hopfield, asked what physics itself can compute and where the thermodynamic limits of computation lie (Feynman, 1982, 1996). What these pioneers of physics and computing intended was not the imitation of neurons but the grounding of computation in physical dynamics and energy. Physics of Computation is a field that flourished in the mid-1990s and has since morphed into “Unconventional Computing,” an all-encompassing name and research field, one that certainly includes neuromorphic computing.

This editorial addresses a misconception about computation in the current neuromorphic engineering discourse. A computing principle must abstract away from the physical substrate on which it is implemented, an idea introduced by the late (Marr, 1982), and subsequently addressed by Cassidy et al. (2013) separating form and function. Statements such as “uses subthreshold CMOS,” “uses memristors,” or “uses neural processing units” are statements about implementation, i.e., form, not essence, just as “uses transistors” never defined classical computing, and “uses superconducting junctions” does not define quantum computing. If neuromorphic computing is to be a paradigm rather than a product category, its defining principle must survive translation across substrates: silicon, oxides, photonics, or wetware.

What, then, is the substrate-independent essence for neuromorphic computing? The most defensible candidate is “thermodynamic”. Nervous systems evolved under severe metabolic budgets: the human brain runs on roughly 20 watts, and cellular-level energy accounting shows that spiking, signaling, and synaptic transmission are all tuned to minimize metabolic cost (Attwell and Laughlin, 2001; Laughlin et al., 1998). Insect brains sharpen the point. A honeybee forages over kilometers, navigates by landmark and sky compass, communicates, and learns with about a million neurons, and a fruit fly flies, and adapts with roughly a tenth of that, on power budgets in the microwatt-to-milliwatt range. Direct measurements in fly photoreceptors show that the energy-information trade-off is enforced down to the level of single cells (Abshire and Andreou, 2000; Niven et al., 2007). The insect brain is therefore the gold standard for edge-AI embodying full sensing, autonomy, and lifelong learning within a milliwatt power-budget, a benchmark that no current engineered system, neuromorphic or otherwise, comes close to matching. Addressing *function*, neuromorphic computation is thus, at its core, computing with and against thermodynamics: representing problems as energy functions and entropy production, letting physical dynamics perform descent, relaxation, and sampling, and treating statistical mechanics as the native mathematics of the machine. In this sense neuromorphic computing dovetails with the emerging research programs of thermodynamic computing, which take fluctuations, dissipation, and stochastic dynamics as computational primitives rather than nuisances (Hylton et al., 2019; Coles et al., 2023; Chen et al., 2026), and, in particular, with the field of stochastic thermodynamics, where fluctuation theorems, uncertainty relations, and thermodynamic speed limits now make it possible to quantify the energy costs of computation in systems arbitrarily far from equilibrium, from digital circuits to brains (Wolpert et al., 2024). Indeed, it is now known that any constraint, whether structural, architectural, or physical, produces entropy or dissipates energy. Therefore, the essence of neuromorphic computing could be “thermodynamic” mechanisms that optimize parallel processing under physical constraints of energy, delay, and structural variability (Cassidy et al., 2013; Gangopadhyay and Chakrabartty, 2021).

So what roadmap should any thermodynamics-based neuromorphic framework follow? First, to gain acceptance, such a thermodynamics-based framework would first need to explain the four principles that many in the field currently consider the fundamental signature and starting point of neuromorphic computing: *(a)* Event-based, asynchronous representation, where computation, sensing, encoding and communication is unified (Boahen, 2022; Gangopadhyay et al., 2020) and information is carried by the timing of sparse events rather than by clocked global state (Purohit and Manohar, 2022; Zhou et al., 2025; Srivatsav et al., 2023); *(b)* Distributed and massive parallelism, with memory and processing collocated (Pouliquen et al., 1997; Andreou et al., 2026) within millions to billions of simple interconnected units (Cauwenberghs, 2013). *(c)* Analog processing, which extracts precision per joule from continuous representation that exploits device physics and locality (Sarpeshkar, 1998); and most importantly *(d)* Noise-induced transitions and fluctuations, in which thermal and device noise and possibly quantum fluctuations drive escape from local minima, probabilistic inference and stochastic computation (Mendat et al., 2015), and exploration, making noise a computational resource rather than an error to be suppressed (Buesing et al., 2011; Chen et al., 2025).

Architecturally, the framework should then provide a scientific rationale for building machines based on multi-time-scale dynamics: fast membrane-like state variables, slower synaptic traces, and still slower homeostatic and structural processes, all coupled through local, continual learning rules and network oscillations. In such a machine there is no distinction between inference and learning. Both are the same physical dynamics viewed at different time scales, with no separate training phase, and no offline weight download. Consequently, everything observed and measured at the system level like polychronicity, or predictive and efficient coding are emergent phenomena. Synchronization across modular units, for instance, is not programmed but arises from coupling and possibly due to constraints on system entropy production, in the way oscillatory assemblies emerge across columns of cortex (Hopfield, 1982; Gray et al., 1989). Attention, likewise, is not a scheduled control signal but an emergent allocation policy, guided by the need to improve system-level energy efficiency by concentrating the metabolic budget (sparse activity, selective routing, selective plasticity) to where information matters most (Lennie, 2003).

To be practically meaningful, any thermodynamics-based framework and its underlying design principles must be scalable. While a dynamical system with a few hundred analog units remains a laboratory curiosity, the brain provides an existence proof scaling to over 80 billion neurons. Scaling to this magnitude demands overcoming device mismatch, wiring constraints, thermal budgets, and the absence of global clocks or error signals. These challenges are fundamental research problems rather than mere downstream engineering details (Kudithipudi et al., 2025; Mehonic and Kenyon, 2022). Crucially, spatial scale is only one dimension of this challenge. Beyond traditional metrics such as number of state variables, neurons, synapses, dendrites, or astrocytes, scaling must also encompass functional diversity (Cauwenberghs, 2013). A fruit fly brain, containing just 100,000 neurons and 50 million synapses, exhibits an extraordinarily rich functional repertoire, a hallmark signature of “general intelligence”. In contrast, neuromorphic computing and mainstream AI remain focused on scaling unit count alone, pushing beyond a trillion parameters in pursuit of artificial general intelligence (AGI). As the exorbitant energy demands of this approach expose the limits of current AI roadmaps (Chen et al., 2026), the field will inevitably need to revisit and adopt neurobiological forms which includes modularity, 2.5D/3D integration (Marwick and Andreou, 2006), and hierarchy, all grounded in “thermodynamic” principles (Wolpert et al., 2024).

Finally, the hope is that the framework will impose upon the neuromorphic paradigm the same discipline that quantum computing imposed on itself: a clearly articulated neuromorphic advantage. Quantum researchers are expected to identify problems where quantum mechanics provably or plausibly beats the best classical alternative (Arute et al., 2019); neuromorphic researchers should be held to the same standard against GPUs or Graphical Processing Units. It is anticipated that such an advantage will arise from efficient and genuinely new ways of exploiting thermodynamics, fluctuations and noise, from the efficient physical implementation of large-scale stochastic and randomized algorithms (Ackley et al., 1985; Theilman and Aimone, 2025; Chen et al., 2025; Ahsan et al., 2026; Nikhar et al., 2024): sampling, annealing, Monte Carlo inference, stochastic optimization, and randomized linear algebra, executed by physics at thermodynamic cost rather than simulated at digital cost. This standard will force a distinction the field has been reluctant to make: neuromorphic *emulation* vs. neuromorphic *realization*. Emulation reproduces neural dynamics (spikes, plasticity, asynchrony) on top of standard synchronous digital logic; realization lets the physics of the substrate itself carry the computation. Because scaling analog dynamics is hard and because the sources of neuromorphic advantage are not yet understood, most current platforms, including the largest spiking systems, are emulators built from digital circuits (Merolla et al., 2014; Davies et al., 2018; Furber et al., 2014). Emulation is genuinely useful: it enables algorithm exploration, neuroscience simulation, and low-power edge inference (Blouw et al., 2019). But an emulator pays the digital tax on every spike, and it is far from clear that simulating stochastic analog dynamics with deterministic clocked logic can ever deliver a decisive advantage over GPUs that are themselves superb digital simulators. If the field settles for emulation, it risks becoming an accelerator niche. If it pursues realization, that is, computing with energy, dynamics, and noise as the brain does, it may yet become what Mead, Hopfield, and Feynman glimpsed: a third computing paradigm, with “thermodynamics” as its core logic.
